# Pharmaceutical Processes

**Published:** 1801-07

**Authors:** 


### *Pharmaceutical Processes.*

SCHMIDT's *ſhort and easy method of preparing the Æthiops antimonialis.*—The common proceeding, according to which the quickſilver is combined with the antimony by trituration, being very long and tedious, Mr. Schmidt endeavoured to find out a ſhorter way of obtaining that preparation, which is the following. Half an ounce of pure quickſilver is mixed with one drachm of ſublimed ſulphur in a ſmall crucible, which is put into hot aſhes, and the mixture continually ſtirred till the ſulphur is melted. The mixture became ſoon grey, but as the globules did not yet diſappear, half a drachm more of ſulphur was added, and being melted, the quickſilver totally diſappeared, and the whole mixture became grey and homogeneous. After it had been ground in a ſtone mortar, one ounce of antimonium crudum was added; and in this way an Æthiops antimonialis was obtained in the ſpace of three hours, in every reſpect equal to that which is prepared by mere trituration in the ſpace of ſome days.

---

BOUILLON-LAGRANGE's *method of preparing the ſyrup of quickſilver.*—This mercurial preparation was firſt invented by Mr. BELET, and is much recommended by PORTAL. There are ſeveral methods of preparing it, all which, however, afford a very imperfect preparation, on account of the quickſilver ſeparating itſelf from the mixture, and falling to the bottom of the veſſel when it is kept but a little time. The quickſilver ſyrup of BOUILLON-LAGRANGE, though it agrees on the whole with that of BELET, is yet preferable, becauſe it is not ſo eaſily decompoſed, and may ſtand a long time without producing a precipitation of the quickſilver. It ought never to be taken with any thing warm, as the quickſilver is immediately precipitated in form of a yellowiſh oxyd. The mode of preparation is the following. Prepare a ſolution of pure quickſilver in nitric acid; let the nitrated quickſilver cryſtalliſe, and diſſolve theſe cryſtals once or twice in diſtilled water, by which means a pure ſalt is obtained. Make a ſimple ſyrup, by diſſolving 1 ¼ pound of ſugar in one pint of diſtilled water, and clarify and filtrate this liquor. Diſſolve 93 grains of the above cryſtals of nitrate of quickſilver in a ſufficient quantity of pure diſtilled water. The ſyrup being cold, add to it this ſolution, and then mix in the whole fluid half a drachm of pure nitrous ether. The ſyrup, that is thus prepared, remains perfectly clear ſeveral days.

---

VAN-

VAN-MONS's *method of preparing muriatic ether.*—The difficulties that attend the preparation of muriatic ether, chiefly originate from the oxygen of the muriatic acid acting longer on the alkohol than is neceffary for changing it into ether, by which circumftance an oily mixture is produced. By the proceeding of Citizen VAN-MONS, this difficulty feems to be removed. It is as follows:

Place in a fand bath that is heated by degrees the retort of Woulf's apparatus, which only confifts of a round receiver with a fhort neck and two adopters. Put into it 1,00 of any weight that you choofe of muriate of natron, which is perfectly dry, and pour into the receiver and the two veffels the fame quantity of pure alkohol. The openings being accurately clofed, and the laft adopter fecured by an additional pipe, pour on the falt in the retort 0,50 of concentrated fulphuric acid, and fuffer the operation to proceed in the cold for five or fix hours. Make a gentle fire at firft, but increafe it by degrees. The muriatic acid paffing over in a gafiform ftate, combines itfelf with the alkohol. It is of ufe in this operation to dip the joined pipes into alkohol, which greatly promotes the abforption of the gas. Sometimes it happens, that the alkohol paffes over from one adopter to the other, in which cafe it is requifite to change them, and join that with the receiver which contains the moft alkohol. All muriatic acid being gone over, the liquors of the different veffels are put into the retort, after the falt is taken out. They form a ftrong muriated alkohol. Add now to the liquor 0,20 of oxyd of manganefe, and put into the receiver and the two adopters a quantity of a folution of cauftic kali, and let it diftil carefully by a gentle heat. This alkaline folution is intended to abforb the fuperabundant acid, and to prevent the procefs which makes the ether become a kind of oil. Notwithftanding all poffible care, it cannot be avoided, that a greater or lefs quantity of the ether is decompofed by the acid, and likewife the oxygenated muriatic acid that is combined with the alkali in fome meafure acts there. The ether thus obtained, is mixed with double its weight of diftilled water, and rectified over a gentle fire. A muriatic ether may be obtained by diftilling in the fand-bath a mixture of alkohol and oxygenated muriate of alkali in the proportion of 1,00 to 0,25.

